# Supplementary material for: The Evolution and Characterization of the RNA Interference Pathways in Lophotrochozoa
Source: Genome Biol Evol. 2024 May 7;16(5):evae098. doi: 10.1093/gbe/evae098 (PMC11114477; doi:10.1093/gbe/evae098)
Supplement: evae098_Supplementary_Data [file evae098_supplementary_data.zip › Supplementary_materials.zip!/selected_miRNAs/CrGi_selected_miRNAs/CrGi_NC_047563.1_16607.pdf]

Provisional ID	:	NC_047563.1_16607
Score total	:	16249
Score for star read(s)	:	3.9
Score for read counts	:	16243.7
Score for mfe	:	0.4
Score for randfold	:	1.6
Score for cons. seed	:	-0.6
Total read count	:	31873
Mature read count	:	31856
Loop read count	:	11
Star read count	:	6

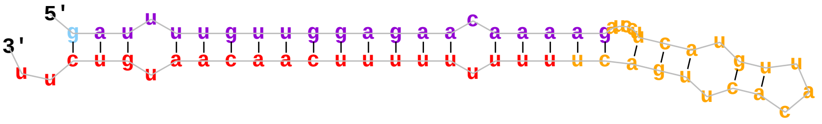

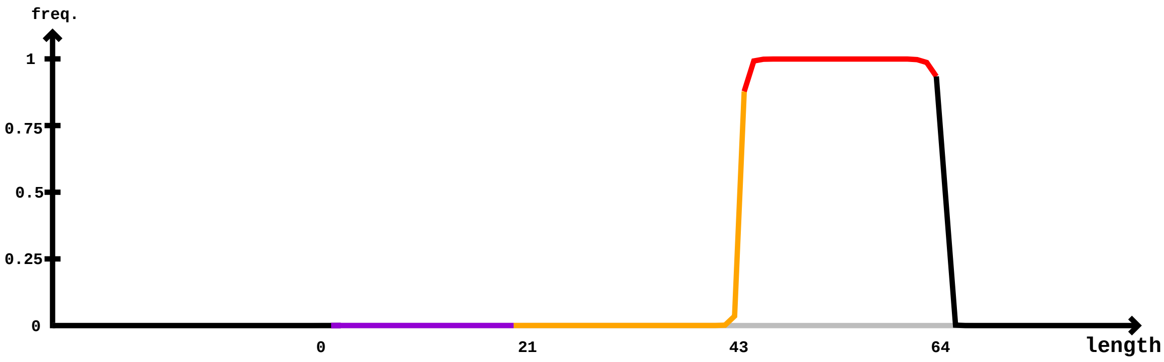


```
                                        Star                                       Mature
5'- agaucuuauucaacucuuuggucucaagauuuuguuggagaacaaaagauccuucauguuacacuugacuuuuuuuuuuucaacaaugucuuuugaccucagggaauagucc  -3'   obs
    agaucuuauucaacucuuuggucucaagauuuuguuggagaacaaaagauccuucauguuacacuugacuuuuuuuuuuucaacaaugucuuuugaccucagggaauagucc         exp
    .............((((..((((..(((((.(((((((((((.(((((.....(((.((...)).)))))))).))))))))))).)))))..))))..)))).........   reads   mm   sample
    .......auucaacucuuuggucuc.......................................................................................   1       0    Mt1
    ..........................agauuuuguuggagaacaaaa.................................................................   1       0    Mt1
    ............................auuuuguuggagaacUaaag................................................................   1       1    Mt1
    ............................auuuuguuggagaacaaaag................................................................   2       0    Mt1
    ................................................auccuucauguuacacuugac...........................................   1       0    Mt1
    .....................................................................Uuuuuuuuuuucaacaauguc......................   1       1    Mt1
    .....................................................................Uuuuuuuuuuucaacaaugucu.....................   1       1    Mt1
    .....................................................................Uuuuuuuuuuucaacaaugucuu....................   9       1    Mt1
    ......................................................................uuuuuuuuuucaacaaugu.......................   1       0    Mt1
    ......................................................................uuuuuuuuuucaacaauguc......................   15      0    Mt1
    ......................................................................uuuuuuuuuucaaUaaugucu.....................   1       1    Mt1
    ......................................................................uuuuuuuuuucaacaaugucu.....................   14      0    Mt1
    ......................................................................Cuuuuuuuuucaacaaugucu.....................   1       1    Mt1
    ......................................................................uuuuuuuuuucaacUaugucuu....................   1       1    Mt1
    ......................................................................uuuuuuuuuucaacaaAgucuu....................   1       1    Mt1
    ......................................................................Cuuuuuuuuucaacaaugucuu....................   7       1    Mt1
    ......................................................................uuuuuuuuuucaacaaugucuu....................   276     0    Mt1
    ......................................................................uuuuuuuuuucaacaaugucuuu...................   36      0    Mt1
    ......................................................................Auuuuuuuuucaacaaugucuuu...................   1       1    Mt1
    ......................................................................uuuuuuuuuucaacaaugucuuuu..................   1       0    Mt1
    .......................................................................uuuuuuuuucaacaaugu.......................   25      0    Mt1
    .......................................................................uuuuuuuuucaacaauguc......................   102     0    Mt1
    .......................................................................Auuuuuuuucaacaaugucu.....................   1       1    Mt1
    .......................................................................uGuuuuuuucaacaaugucu.....................   1       1    Mt1
    .......................................................................uuuuuuuuucaacaaugucu.....................   443     0    Mt1
    .......................................................................Cuuuuuuuucaacaaugucu.....................   1       1    Mt1
    .......................................................................uuuuuuuuucaacaaugCcuu....................   1       1    Mt1
    .......................................................................uuuCuuuuucaacaaugucuu....................   1       1    Mt1
    .......................................................................uuuuuuuuuUaacaaugucuu....................   3       1    Mt1
    .......................................................................uuuuuuuuCcaacaaugucuu....................   1       1    Mt1
    .......................................................................uuuuuuCuucaacaaugucuu....................   1       1    Mt1
    .......................................................................uuuuuuuuucaacaauguUuu....................   7       1    Mt1
    .......................................................................uuuuuuuuucUacaaugucuu....................   1       1    Mt1
```

```
                                   Star                                         Mature
agaucuuauucaacucuuuggucucaagauuuuguuggagaacaaaagauccuucauguuacacuugacuuuuuuuuuucaacaaugucuuuugaccucagggaauagucc
......................................................................uuuuuuuuucaacaaugucuC....................    1      1     Mt1
......................................................................uuuuuuuuucaacaUugucuu....................    1      1     Mt1
......................................................................uuuuuuuCucaacaaugucuu....................    1      1     Mt1
......................................................................Auuuuuuuucaacaaugucuu....................    4      1     Mt1
......................................................................uuuuuCuuucaacaaugucuu....................    1      1     Mt1
......................................................................Guuuuuuuucaacaaugucuu....................    6      1     Mt1
......................................................................uuCuuuuuucaacaaugucuu....................    1      1     Mt1
......................................................................uuuuuuuuucaacaGugucuu....................    1      1     Mt1
......................................................................uuuuuuuuucaacaaugucuu....................    7095   0     Mt1
......................................................................uuuuuuuuucaacaauUucuu....................    5      1     Mt1
......................................................................uuuuuuuuAcaacaaugucuu....................    1      1     Mt1
......................................................................Cuuuuuuuucaacaaugucuu....................    17     1     Mt1
......................................................................uuuuuuuuucaaUaaugucuu....................    2      1     Mt1
......................................................................Guuuuuuuucaacaaugucuuu...................    2      1     Mt1
......................................................................Auuuuuuuucaacaaugucuuu...................    1      1     Mt1
......................................................................uuuuuuuuucaacaaugucCuu...................    1      1     Mt1
......................................................................uuuuuuuuucaacaaugucuuu...................    1041   0     Mt1
......................................................................uuuuuuuCucaacaaugucuuu...................    1      1     Mt1
......................................................................uuuuuuuuucaacaaugucuuC...................    2      1     Mt1
......................................................................uuuuuuuuucaacaaugucuCu...................    1      1     Mt1
......................................................................uuuuuuuuucaacaaugCcuuu...................    1      1     Mt1
......................................................................uuuuuuuuucaacaaugucuuA...................    1      1     Mt1
......................................................................uuuuuuuuucaacaauguUuuu...................    1      1     Mt1
......................................................................uuuuuuuuucaacaaugucuuuA..................    2      1     Mt1
......................................................................uuuuuuuuucaacaaugucuuuu..................    9      0     Mt1
......................................................................uuuuuuuuucaacaaugucuuuAg.................    1      1     Mt1
.......................................................................uuuuuuuucaacaauguc......................    6      0     Mt1
.......................................................................uuuuuuuucaacaaugucu.....................    72     0     Mt1
.......................................................................Auuuuuuucaacaaugucuu....................    1      1     Mt1
.......................................................................Guuuuuuucaacaaugucuu....................    1      1     Mt1
.......................................................................uuuuuuuucaacaaugucuC....................    1      1     Mt1
.......................................................................uuCuuuuucaacaaugucuu....................    1      1     Mt1
.......................................................................uuuuuuuucaacaaugucuu....................    943    0     Mt1
.......................................................................uuuuuuuucaacaauguUuu....................    1      1     Mt1
.......................................................................uuuuuuuucaCcaaugucuu....................    1      1     Mt1
.......................................................................uuuuCuuucaacaaugucuu....................    1      1     Mt1
.......................................................................uuuuuuuucaacaauguGuu....................    1      1     Mt1
.......................................................................Cuuuuuuucaacaaugucuu....................    1      1     Mt1
.......................................................................uuuCuuuucaacaaugucuuu...................    1      1     Mt1
.......................................................................uuCuuuuucaacaaugucuuu...................    1      1     Mt1
.......................................................................uuuuuuuucaacaauUucuuu...................    1      1     Mt1
.......................................................................uuuuuuuucaacaaugucuuu...................    153    0     Mt1
.......................................................................uuuuuuuucaacaaugucCuu...................    1      1     Mt1
.......................................................................Cuuuuuuucaacaaugucuuuu..................    1      1     Mt1
........................................................................uuuuuuucaacaaugucu.....................    2      0     Mt1
........................................................................uuuuuuucaacaaugucuu....................    63     0     Mt1
........................................................................uuuuuCucaacaaugucuu....................    1      1     Mt1
........................................................................uuuuuuucaacaaugucuuu...................    13     0     Mt1
.........................................................................uuuuuucaacaaugucuu....................    9      0     Mt1
.........................................................................uuuuuucaacaaugucuuu...................    2      0     Mt1
..........................................................................uuuuucaacaaugucuuu...................    1      0     Mt1

..........................agauuuuguuggagaacaaaa................................................................    1      0     Mt2
..........................agauuuuguuggagaacaaaag...............................................................    1      0     Mt2
................................................auccuucauguuacacuuga...........................................    1      0     Mt2
................................................auccuucauguuacacuugac..........................................    5      0     Mt2
.................................................uccuucauguuacacuuga...........................................    2      0     Mt2
....................................................................cuuuuuuuuuucaacaauguc......................    1      0     Mt2
....................................................................Uuuuuuuuuuucaacaaugucuu....................    10     1     Mt2
....................................................................Uuuuuuuuuuucaacaaugucuuu...................    1      1     Mt2
.....................................................................uuuuuuuuuucaacaaugu.......................    1      0     Mt2
.....................................................................uuuuuuuuuucaacaauguc......................    18     0     Mt2
.....................................................................uuuuuuuuuucaaUaaugucu.....................    1      1     Mt2
.....................................................................uuuuuuuuuucaacaaugucu.....................    25     0     Mt2
.....................................................................uuuuuuuuuucaacaaugucuu....................    283    0     Mt2
.....................................................................Cuuuuuuuuucaacaaugucuu....................    12     1     Mt2
.....................................................................Guuuuuuuuucaacaaugucuu....................    1      1     Mt2
.....................................................................uuuuuuuuuucaCcaaugucuu....................    1      1     Mt2
.....................................................................uuuuuuuuuucaacaaugucuuu...................    40     0     Mt2
.....................................................................uuuuuuuuuucaacaauCucuuu...................    1      1     Mt2
```

Star Mature

agaucuuauucaacucuuuggucucaagauuuuguuggagaacaaaagauccuucauguuacacuugacuuuuuuuuucaacaaugucuuuugaccucagggaauagucc

....uuuuuuuuucaacaaugu.......................	23	0	Mt2
....uuuuuuuuucaacaauguc......................	85	0	Mt2
....uuuuuuuuucaacaaugucu.....................	496	0	Mt2
....Auuuuuuuucaacaaugucu.....................	1	1	Mt2
....uuuuuuuuucaacaauUucu.....................	1	1	Mt2
....Cuuuuuuuucaacaaugucu.....................	1	1	Mt2
....Guuuuuuuucaacaaugucu.....................	1	1	Mt2
....uuCuuuuuucaacaaugucuu....................	7	1	Mt2
....uuuuuuuuucaacaauguUuu....................	8	1	Mt2
....uuuuuuuuucGacaaugucuu....................	1	1	Mt2
....uuuuuGuuucaacaaugucuu....................	1	1	Mt2
....uuuuuuuuucaaGaaugucuu....................	1	1	Mt2
....uuuuuuuuuUaacaaugucuu....................	4	1	Mt2
....uuuCuuuuucaacaaugucuu....................	2	1	Mt2
....uuuuuuuCucaacaaugucuu....................	1	1	Mt2
....uuuuuuuuucaacaaugGcuu....................	2	1	Mt2
....uuuuuuuuucaacGaugucuu....................	1	1	Mt2
....uuuuuuuuucaacaaugucuC....................	1	1	Mt2
....uuuuuuuuucaaUaaugucuu....................	3	1	Mt2
....Auuuuuuuucaacaaugucuu....................	11	1	Mt2
....uuuuuuuuucaacaauUucuu....................	6	1	Mt2
....uuuuuuuuuGaacaaugucuu....................	1	1	Mt2
....uuuuuuuuucaacaaugucuG....................	1	1	Mt2
....uuuuuuuuucaacaCugucuu....................	2	1	Mt2
....uuuuuuuuucaaAaaugucuu....................	1	1	Mt2
....uuuuuuuuucaacaGugucuu....................	2	1	Mt2
....uuuuuCuuucaacaaugucuu....................	1	1	Mt2
....Cuuuuuuuucaacaaugucuu....................	19	1	Mt2
....uuuuuuuuucaacaaugucuu....................	7794	0	Mt2
....uuuuuuuGucaacaaugucuu....................	1	1	Mt2
....Guuuuuuuucaacaaugucuu....................	6	1	Mt2
....uuuuuuuuucaacaaCgucuu....................	1	1	Mt2
....uuAuuuuuucaacaaugucuu....................	1	1	Mt2
....uuuuuuuuucaCcaaugucuu....................	2	1	Mt2
....uuuuuuuuucaacaaugucuuu...................	1110	0	Mt2
....Guuuuuuuucaacaaugucuuu...................	1	1	Mt2
....uuuuuuuuucaacaauguUuuu...................	1	1	Mt2
....uuuuuuuuuUaacaaugucuuu...................	2	1	Mt2
....Cuuuuuuuucaacaaugucuuu...................	5	1	Mt2
....Auuuuuuuucaacaaugucuuu...................	2	1	Mt2
....uuuuuuuuucaacaaugAcuuu...................	1	1	Mt2
....uuuuuuuuucaacaaugucuuuA..................	2	1	Mt2
....uuuuuuuuucaacaaugucuuuu..................	8	0	Mt2
....uuuuuuuuucaacaaugucGuuu..................	1	1	Mt2
....uuuuuuuucaacaauguc......................	10	0	Mt2
....uuuuuuuucaacaaugucu.....................	65	0	Mt2
....Guuuuuuucaacaaugucuu....................	5	1	Mt2
....uuuuuCuucaacaaugucuu....................	1	1	Mt2
....uuuuuuuucaGcaaugucuu....................	1	1	Mt2
....uuuuuuuuUaacaaugucuu....................	2	1	Mt2
....uuuuuuuucaacaaugucuu....................	1076	0	Mt2
....Cuuuuuuucaacaaugucuu....................	2	1	Mt2
....uuuuuuuucCacaaugucuu....................	1	1	Mt2
....Auuuuuuucaacaaugucuu....................	2	1	Mt2
....uuuuuuuucaacaauguUuu....................	1	1	Mt2
....uuuuuuuucaacaaugucuuu...................	151	0	Mt2
....Guuuuuuucaacaaugucuuu...................	1	1	Mt2
....uuuuuuuucaacaaugucuuuu..................	5	0	Mt2
....uuuuuuuucaacaaugucuuuuU.................	1	1	Mt2
....uuuuuuucaacaaugucu.....................	2	0	Mt2
....uuuuuuucaacaaugucuu....................	60	0	Mt2
....uuuuuuucaacaauguUuu....................	1	1	Mt2
....uuuuuuucaacaaugucuuu...................	9	0	Mt2
....uuuuuucaacaaugucuu....................	7	0	Mt2
....Uuuuuuuuuuucaacaauguc......................	1	1	Ov2
....Uuuuuuuuuuucaacaaugucuu....................	5	1	Ov2
....uuuuuuuuuuucaacaaugu.......................	2	0	Ov2
....uuuuuuuuuuucaacaauguc......................	9	0	Ov2
....Cuuuuuuuuucaacaaugucu.....................	2	1	Ov2

```
                              Star                                        Mature
agaucuuauucaacucuuuggucucaagauuuuguuggagaacaaaagauccuucauguuacacuugacuuuuuuuuuucaacaaugucuuuugaccucagggaauagucc
.....................................................................uuuuuuuuuucaacaaugucu.....................    13     0   Ov2
.....................................................................Cuuuuuuuuucaacaaugucuu....................    5      1   Ov2
.....................................................................uuuuuuuuuucaacaaugucuu....................    134    0   Ov2
.....................................................................uuuuuuuuuucaacaaugucuuu...................    22     0   Ov2
.....................................................................uuuuuuuuuuUaacaaugucuuu...................    2      1   Ov2
.....................................................................uuuuuuuuuucaacaaugucuuuA..................    1      1   Ov2
......................................................................uuuuuuuuucaacaaugu.......................    11     0   Ov2
......................................................................uuuuuuuuucaacaauguc......................    40     0   Ov2
......................................................................Auuuuuuuucaacaaugucu.....................    1      1   Ov2
......................................................................uuuuuuuuucaacaaugGcu.....................    1      1   Ov2
......................................................................uuuuuuuuucaacaaugucu.....................    212    0   Ov2
......................................................................Cuuuuuuuucaacaaugucu.....................    1      1   Ov2
......................................................................Cuuuuuuuucaacaaugucuu....................    6      1   Ov2
......................................................................Auuuuuuuucaacaaugucuu....................    7      1   Ov2
......................................................................Guuuuuuuucaacaaugucuu....................    4      1   Ov2
......................................................................uuuuGuuuucaacaaugucuu....................    1      1   Ov2
......................................................................uCuuuuuuucaacaaugucuu....................    1      1   Ov2
......................................................................uuuGuuuuucaacaaugucuu....................    1      1   Ov2
......................................................................uuuuuuuCucaacaaugucuu....................    1      1   Ov2
......................................................................uuuuuuuuucaacaaugGcuu....................    1      1   Ov2
......................................................................uuuuuuuuucCacaaugucuu....................    1      1   Ov2
......................................................................uuuuuuuuucaaUaaugucuu....................    1      1   Ov2
......................................................................uuuuuuuuucaacaaugucuu....................    3646   0   Ov2
......................................................................uuuuuCuuucaacaaugucuu....................    1      1   Ov2
......................................................................uuCuuuuuucaacaaugucuu....................    1      1   Ov2
......................................................................uuuuuuuuucaacaauUucuu....................    4      1   Ov2
......................................................................uuuuuuuuucaacUaugucuu....................    1      1   Ov2
......................................................................uuuuuuuuucaacaauguUuu....................    4      1   Ov2
......................................................................uuuuuuuuucaacaCugucuu....................    1      1   Ov2
......................................................................Auuuuuuuucaacaaugucuuu...................    1      1   Ov2
......................................................................uuuuuCuuucaacaaugucuuu...................    1      1   Ov2
......................................................................uuuuuuuuucaacaaugucuuu...................    396    0   Ov2
......................................................................uuuuuuuuucaacaauguUuuu...................    1      1   Ov2
......................................................................uuuuuuuuucGacaaugucuuu...................    1      1   Ov2
......................................................................uuAuuuuuucaacaaugucuuu...................    1      1   Ov2
......................................................................uCuuuuuuucaacaaugucuuu...................    1      1   Ov2
......................................................................uuuuuuuuucaacaaugucuuuu..................    7      0   Ov2
......................................................................Cuuuuuuuucaacaaugucuuuu..................    1      1   Ov2
......................................................................uuuuuuuuucaacaaugucuuuAg.................    1      1   Ov2
......................................................................uuuuuuuuucaacaaugucuuuuU.................    1      1   Ov2
.......................................................................uuuuuuuucaacaauguc......................    2      0   Ov2
.......................................................................uuuuuuuucaacaaugucu.....................    41     0   Ov2
.......................................................................Cuuuuuuucaacaaugucuu....................    2      1   Ov2
.......................................................................uuuuuuuucaacaaugucuC....................    1      1   Ov2
.......................................................................Auuuuuuucaacaaugucuu....................    1      1   Ov2
.......................................................................uuuuuuuucaCcaaugucuu....................    1      1   Ov2
.......................................................................uuCuuuuucaacaaugucuu....................    1      1   Ov2
.......................................................................uuuuuuuucaacaaugucuu....................    491    0   Ov2
.......................................................................Guuuuuuucaacaaugucuu....................    2      1   Ov2
.......................................................................uuuCuuuucaacaaugucuu....................    1      1   Ov2
.......................................................................uuuuuuuucaacaaugucuuu...................    45     0   Ov2
.......................................................................uuuuuuuucaacaaugucuuuu..................    1      0   Ov2
........................................................................uuuuuuucaacaaugucuu....................    21     0   Ov2
........................................................................uuuuuuucaacaauguUuuu...................    1      1   Ov2
........................................................................uuuuuuucaacaaugucuuu...................    5      0   Ov2
.........................................................................uuuuuucaacaaugucuu....................    1      0   Ov2

................................................auccuucauguuacacuuga...........................................    1      0   Ov1
................................................auccuucauguuacacuugac..........................................    1      0   Ov1
....................................................................Uuuuuuuuuuucaacaauguc......................    1      1   Ov1
....................................................................cuuuuuuuuuucaacaauguc......................    1      0   Ov1
....................................................................Uuuuuuuuuuucaacaaugucuu....................    7      1   Ov1
....................................................................Uuuuuuuuuuucaacaaugucuuu...................    1      1   Ov1
.....................................................................uuuuuuuuuucaacaaugu.......................    1      0   Ov1
.....................................................................uuuuuuuuuucaacaauguc......................    2      0   Ov1
.....................................................................uuuuuuuuuucaacaaugucu.....................    17     0   Ov1
.....................................................................Cuuuuuuuuucaacaaugucuu....................    4      1   Ov1
.....................................................................uuuuuuuuuucaacaaugucuu....................    117    0   Ov1
.....................................................................uuuuuuuuuucaacaaAgucuu....................    1      1   Ov1
.....................................................................uuuuuuuGuucaacaaugucuu....................    1      1   Ov1
```

```
                            Star                                      Mature
agaucuuauucaacucuuuggucucaagauuuuguuggagaacaaaagauccuucauguuacacuugacuuuuuuuuuucaacaaugucuuuugaccucagggaauagucc
.....................................................................uuuuuuuuuucaacaauguUuu.................... 1     1  Ov1
.....................................................................uuuuuuuuCucaacaaugucuu.................... 1     1  Ov1
.....................................................................uuuuuuuuuucaacaaugucuuu................... 18    0  Ov1
.....................................................................uuuuuuuuuuUaacaaugucuuu................... 1     1  Ov1
.....................................................................uuuuuuuuuucaacaaugucuuuu.................. 1     0  Ov1
......................................................................uuuuuuuuucaacaaugu....................... 8     0  Ov1
......................................................................uuuGuuuuucaacaauguc...................... 1     1  Ov1
......................................................................Auuuuuuuucaacaauguc...................... 1     1  Ov1
......................................................................uuuuuuuuucaacaauguc...................... 36    0  Ov1
......................................................................Guuuuuuuucaacaauguc...................... 2     1  Ov1
......................................................................uuuuuuuuucaacaaugucu..................... 217   0  Ov1
......................................................................Cuuuuuuuucaacaaugucu..................... 1     1  Ov1
......................................................................uuuuuuuuucaacaauUucuu.................... 2     1  Ov1
......................................................................uuuuuuuuucaacaauguUuu.................... 2     1  Ov1
......................................................................uuuuuuuuucaacaaugCcuu.................... 1     1  Ov1
......................................................................uuCuuuuuucaacaaugucuu.................... 5     1  Ov1
......................................................................uuuuuuuuucaacaaugucuC.................... 1     1  Ov1
......................................................................uuuuuuuuucaacaaugAcuu.................... 2     1  Ov1
......................................................................uuuuGuuuucaacaaugucuu.................... 1     1  Ov1
......................................................................Auuuuuuuucaacaaugucuu.................... 6     1  Ov1
......................................................................uuuuuuuuuUaacaaugucuu.................... 1     1  Ov1
......................................................................Cuuuuuuuucaacaaugucuu.................... 8     1  Ov1
......................................................................uuuuuuuuucaGcaaugucuu.................... 1     1  Ov1
......................................................................uuuuuuuuucaacaCugucuu.................... 1     1  Ov1
......................................................................uuuuuuuuucaacaaugucuu.................... 3379  0  Ov1
......................................................................uuuuuuuuucaacaUugucuu.................... 1     1  Ov1
......................................................................Guuuuuuuucaacaaugucuu.................... 4     1  Ov1
......................................................................uuuuuuuuucaacaaugGcuuu................... 1     1  Ov1
......................................................................uCuuuuuuucaacaaugucuuu................... 1     1  Ov1
......................................................................uuuuuuuuucaacaaugucuuu................... 395   0  Ov1
......................................................................uuuuuuuuucaacaaugucuuuu.................. 4     0  Ov1
.......................................................................uuuuuuuucaacaauguc...................... 6     0  Ov1
.......................................................................uuuuuuuucaacaaugucu..................... 30    0  Ov1
.......................................................................uuuuuuuucaacaaugucuu.................... 439   0  Ov1
.......................................................................Guuuuuuucaacaaugucuu.................... 1     1  Ov1
.......................................................................Cuuuuuuucaacaaugucuu.................... 1     1  Ov1
.......................................................................uuuuuuuucaacaaugucuuu................... 61    0  Ov1
.......................................................................uuuuuuuucaacaaugucuuuu.................. 2     0  Ov1
........................................................................uuuuuuucaacaaugucu..................... 1     0  Ov1
........................................................................uuuuuuucaacaaugucuu.................... 25    0  Ov1
........................................................................uuuuuuucaacaaugucuuu................... 3     0  Ov1
.........................................................................uuuuuucaacaaugucuu.................... 4     0  Ov1
```
